# Severe weight loss after minimally invasive oesophagectomy is associated with poor survival in patients with oesophageal cancer at 5 years

**DOI:** 10.1186/s12876-020-01543-1

**Published:** 2020-12-03

**Authors:** Yasufumi Koterazawa, Taro Oshikiri, Gosuke Takiguchi, Naoki Urakawa, Hiroshi Hasegawa, Masashi Yamamoto, Shingo Kanaji, Kimihiro Yamashita, Takeru Matsuda, Tetsu Nakamura, Satoshi Suzuki, Yoshihiro Kakeji

**Affiliations:** Division of Gastrointestinal Surgery, Department of Surgery, Kobe University Graduate School of Medicine, 7-5-2, Kusunoki-cho, Chuo-ku, Kobe City, Hyogo 650-0017 Japan

**Keywords:** Severe weight loss, Minimally invasive oesophagectomy, Enteral nutrition

## Abstract

**Background:**

Patients often experience severe weight loss after oesophagectomy. Enteral nutrition via a feeding jejunostomy tube (FT) is commonly practised. This study aimed to assess the effect of severe weight loss postoperatively and enteral nutrition via an FT on long-term prognosis after oesophagectomy.

**Methods:**

This study analysed 317 patients who underwent minimally invasive oesophagectomy at Kobe University Hospital and Hyogo Cancer Center from 2010 to 2015. The patients’ body weight was evaluated at 3 months postoperatively. They were organised into the severe weight loss (n = 65) and moderate weight loss (n = 252) groups. Furthermore, they were categorised into the FT group (184 patients who had an FT placed during oesophagectomy) and no-FT group (133 patients without FT). Patients (119 per group) matched for the FT and no-FT groups were identified via propensity score matching.

**Results:**

The 5-year overall survival (OS) rate in the severe weight loss group was significantly lower (*p* = 0.024). In the multivariate analysis, tumour invasion depth (pT3-4), preoperative therapy and severe weight loss had a worse OS (hazard ratio = 1.89; 95% confidence interval = 1.12–3.17, hazard ratio = 2.11; 95% confidence interval = 1.25–3.54, hazard ratio = 1.82; 95% confidence interval = 1.02–3.524, respectively). No significant differences in the number of severe weight loss patients and OS were found between the FT and no-FT groups.

**Conclusion:**

Severe weight loss is significantly associated with poor OS. In addition, enteral nutrition via an FT did not improve the severe weight loss and OS.

## Background

Oesophageal cancer is the eighth most common cancer worldwide [1]. Oesophagectomy is one of the main procedures of treatment for oesophageal cancer; however, compared with other gastroenterological surgeries, it is a highly invasive procedure [2]. After oesophagectomy, many patients have reduced appetite and oral intake, which lead to severe weight loss [3]. Postoperative malnutrition is associated with poor prognosis in patients with oesophageal cancer [4–6].

We recently performed minimally invasive oesophagectomy (MIO). MIO improves not only postoperative complications [7] but also postoperative nutritional status, including serum albumin levels [8], appetite, and body mass index (BMI) [9]. However, the effect of severe weight loss after MIO on long-term prognosis has been poorly investigated.

Moreover, enteral nutrition for patients who had undergone a major gastrointestinal surgery improves the postoperative nutritional status and decreases postoperative complications [10, 11]. Enteral nutrition via a feeding jejunostomy tube (FT) is commonly practised after oesophagectomy and recommended by programs on enhanced recovery after surgery [12]. However, no studies have assessed whether these nutritional interventions can improve severe weight loss and poor prognosis after oesophagectomy.

Therefore, this retrospective study aimed to assess the effect of severe weight loss postoperatively and enteral nutrition via an FT during thoracoscopic oesophagectomy on long-term prognosis after MIO.

## Methods

### Aim and objective

We wanted to determine whether severe weight loss after MIO was associated with overall survival outcomes at 5-year post-operative follow up in patients with oesophageal cancer, by collecting data from patients who underwent MIO in Kobe University Hospital and Hyogo Cancer Center. We investigated the following variables (age, gender, pathological depth of tumor invasion, pathological lymph node metastasis, residual tumor, preoperative therapy, severe weight loss, anastomosis leakage, pulmonary complication and recurrent nerve palsy) using multivariate analysis.

### Patient population

From April 2010 to December 2015, 361 patients who had developed thoracic oesophageal cancer underwent MIO at Kobe University Hospital and Hyogo Cancer Center. Subsequently, some cases were excluded owing to salvage oesophagectomy (n = 15), mortality within 3 months (n = 12), and lack of clinical data (n = 17); thus, a total of 317 patients were enrolled in this study.

First, to assess the effect of postoperative weight loss on the patients’ long-term prognosis, patients were classified into the severe weight loss and moderate weight loss groups. We compare the overall survival (OS), cancer-specific survival (CSS), and progression-free survival (PFS) rates at 5 years in the severe and moderate weight loss groups. In addition, multivariate analysis was conducted to assess whether postoperative weight loss is associated with poor overall survival. In assessing the effect of enteral nutrition via an FT during oesophagectomy, patients who had an FT placed during oesophagectomy were categorised into the FT group, whereas those without the FT were categorised into the no-FT group. Then, the number of patients with postoperative severe weight loss and the OS in the matched cohort were compared.

Enhanced computed tomography (CT) and endoscopy were performed for staging. The clinical stage was decided based on the seventh edition of the tumour–node–metastasis classification established by the Union for International Cancer Control [13]. Neoadjuvant chemotherapy (two cycles of cisplatin and 5-FU) was administered to patients with cT2-4 or patients clinically positive for lymph node metastasis [14]. Before oesophagectomy, patients with obstructing oesophageal carcinoma were administered oral nutritional supplements via a nasojejunal tube, which was endoscopically placed for enteral nutrition.

### Surgical procedure

All study patients underwent thoracoscopic oesophagectomy with total mediastinal lymphadenectomy in the prone position, as described previously [15–17]. The abdominal procedure was performed either laparoscopically or as an open laparotomy. Initially, gastric mobilisation was performed, followed by abdominal lymphadenectomy. Then, a 3–4-cm gastric conduit was created, and it was raised via the posterior mediastinum.

Subsequently, the surgeons inserted a Kangaroo™ 9-Fr jejunostomy catheter into the jejunum using the Stamm technique for postoperative enteral nutrition. The two affiliated institutions have a uniform therapeutic strategy. There were no criteria about FT placement. FT placement was decided by the attending surgeon, and the decision depended on patient age, comorbidities, clinical stage, and performance of preoperative therapy. FT was likely placed in patients with deep tumour invasion who underwent preoperative therapy.

### Evaluation of postoperative clinical course

A jejunostomy catheter was placed during oesophagectomy, as described above. In the FT group, continuous enteral nutrition was commenced within 48 h after oesophagectomy. The patients received an elemental diet (ELENTAL®, EA Pharma, Tokyo, Japan). The initial administration rate was 20 mL/h; this was increased by 20 mL/h every 2 days to 80 mL/h. Then, the elemental diet was changed to an oligomeric formula (Twinline-NF®, Otsuka, Tokyo, Japan). After the confirmation of the absence of recurrent laryngeal nerve palsy and anastomotic leakage on postoperative day 7 by an otorhinolaryngologist, oral intake was commenced and enteral feeding was reduced to 400 mL/h. Enteral nutrition was continued after hospital discharge until patients could consume an adequate amount of calories orally.

Body weight was measured twice during the study, that is, preoperatively at the time of surgery and 3 months after surgery. The optimal cutoff value was determined by a minimum *p*-value approach [18, 19]. The severe weight loss cutoff point for the OS was 17%, with the χ^2^ log-rank value of 4.93 (*p* = 0.026). Furthermore, patients were organised into the severe weight loss group and moderate weight loss group.

### Propensity score matching

Propensity score matching was employed for assembling two comparable groups [20]. The propensity score of patients in the FT group was estimated, and each patient was matched to those in the no-FT group with the closest propensity score, using a simple 1:1 nearest-neighbour matching algorithm. A calliper of 0.20 of the standard deviation of the logit in the propensity score was imposed. The following covariates were used for propensity score-matched analysis: age, sex, tumour invasion depth, lymph node metastasis, and preoperative therapy. Initially, the FT group was composed of 184 patients and the no-FT group of 133 patients; after the propensity score-matched analysis, each group had 119 patients, accounting for 238 in total.

### Statistical analysis

Patient’s data on clinical and pathological Stage, body weight, and clinical outcomes were collected from their medical charts. By using the Clavien–Dindo classification, complications were defined and were subsequently recorded [21]. The variables under investigation are as follows: age, gender, location of tumor, clinical and pathological depth of tumor invasion, clinical and pathological lymph node metastasis, residual tumor, preoperative therapy, severe weight loss, anastomosis leakage (Clavien–Dindo 3 and 4), pulmonary complication (Clavien–Dindo 3 and 4) and recurrent nerve palsy (Clavien–Dindo 3 and 4). Differences between variables were analysed using Student’s *t* test, χ^2^ test, or Cox proportional hazard model, as appropriate. All analyses were performed on JMP® 10 (SAS Institute Inc., Cary, NC, USA), and *p* < 0.05 indicates significance.

## Results

### Distribution of weight loss at 3 months postoperatively.

**Fig. 1:**
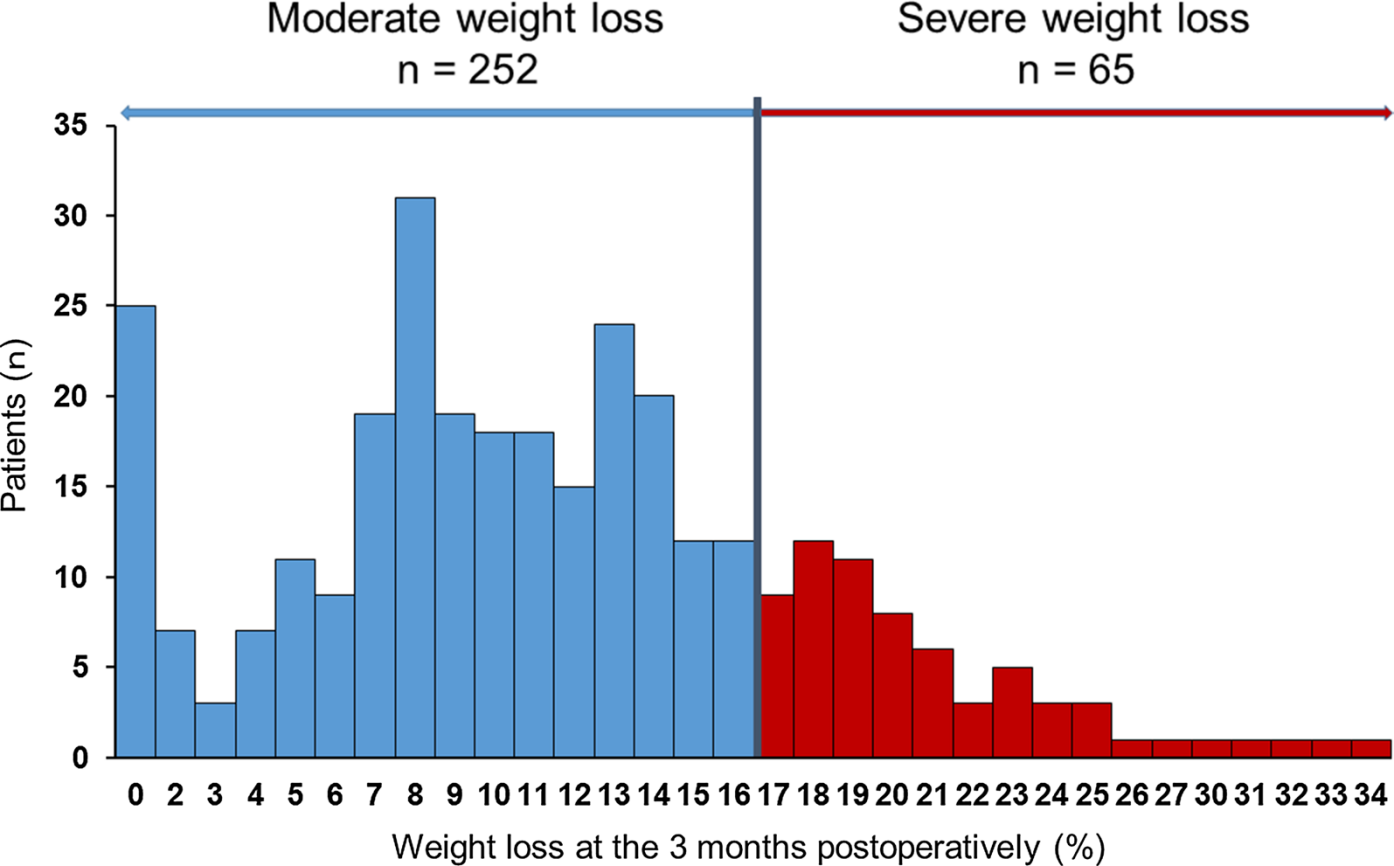
Distribution of weight loss at 3 months postoperatively

The distribution of weight loss values at 3 months postoperatively is shown in Fig. 1. The patients categorised into a severe weight loss group (n = 65) and a moderate weight loss group (n = 252).

### Comparison of clinical features between the severe weight loss and moderate weight loss groups

Clinical features were compared between patients with severe weight loss (≥ 17%: 65 patients) and those with moderate weight loss (< 17%: 252 patients). The demographic and clinical characteristics of the groups are summarised in Table 1. The two groups were comparable in terms of age, sex, tumour location, clinical and pathological tumour invasion depth, clinical and pathological lymph node metastasis, residual tumor, FT placement, preoperative body weight, anastomotic leakage incidence, frequency of recurrent laryngeal nerve palsy, and pulmonary complication.

**Table 1:** Comparison of clinical features between the severe weight loss and moderate weight loss groups

	Severe weight loss (n = 65)	Moderate weight loss (n = 252)	P value
Age (years)*	68 (50–82)	66 (27–80)	0.15^a^
Gender (%)			0.93^b^
Male	57 (88%)	220 (87%)	
Female	8 (12%)	32 (13%)	
Location of tumor			0.21^b^
Ut/Mt/Lt	8/29/28	53/110/89	
Clinical depth of tumor invasion			0.11^b^
cT1/T2/T3/T4	20/23/22/0	101/55/93/3	
Clinical lymph node metastasis			0.42^b^
cN0/N1/N2/N3	25/32/7/0	125/102/25/1	
Preoperative therapy (+)	42 (65%)	159 (63%)	0.82^b^
pathological depth of tumor invasion			0.65^b^
pT1/T2/T3/T4	33/9/21/2	137/24/87/4	
Pathological lymph node metastasis			0.98^b^
pN0/N1/N2/N3	31/22/8/4	124/79/32/17	
Residual tumor			0.16^b^
R0/R1/R2	57/8/0	226/20/6	
Placement of FJT (+)	39 (60%)	145 (54%)	0.71^b^
Preoperative body weight (kg)*	57 (40–85)	56 (32–83)	0.27^a^
Anastomotic leakage** (+)	5 (7.7%)	30 (12%)	0.32^b^
Pulmonary complication** (+)	4 (6.2%)	14 (5.6%)	0.85^b^
Recurrent nerve palsy** (+)	3 (4.6%)	10 (4.0%)	0.81^b^

*FJT* feeding jejunostomy tube

^*^Data are expressed as the median (range)

^**^Postoperative morbidity was analyzed according to the Clavien–Dindo classification (3 and 4)

^a^χ^2^ test

^b^Student’s *t*-test

### Clinical outcomes in the severe and moderate weight loss groups

**Fig. 2:**
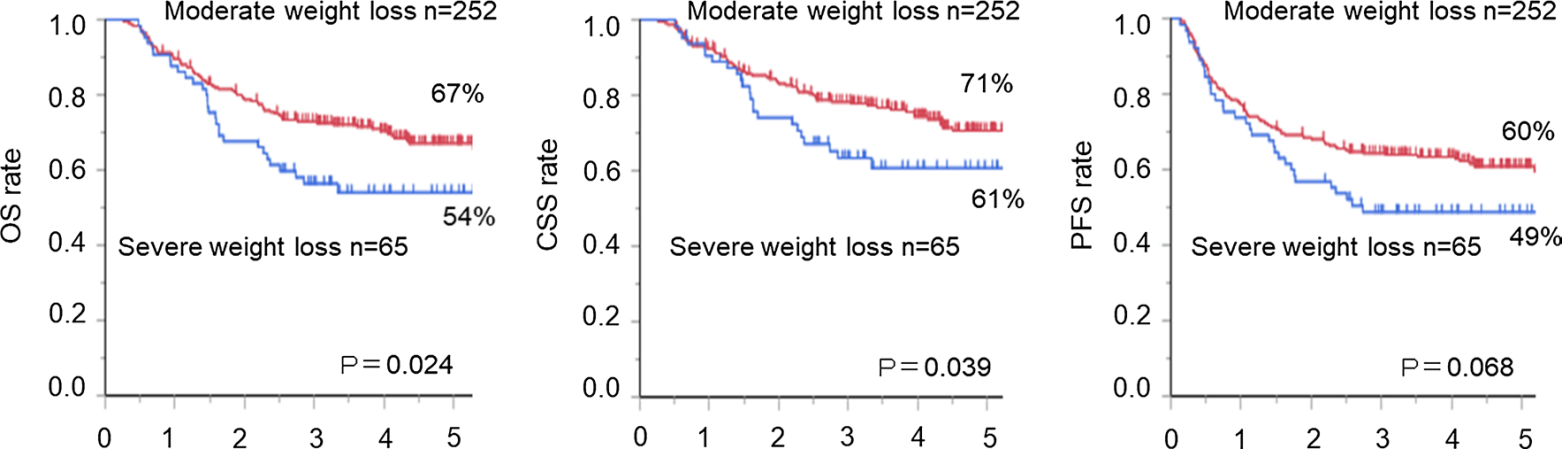
**a** Overall survival (OS) rate in the severe weight loss group (blue line) was significantly lower (*p* = 0.024) than that in the moderate weight loss group (red line). **b** The progression-free survival rates in the severe weight loss group were lower (blue line) than those in the moderate weight loss group (red line), but this difference did not reach significance (*p* = 0.068). **c** The cancer-specific survival rates in the severe weight loss group (blue line) were significantly lower (*p* = 0.039) than those in the moderate weight loss group (red line)

Figure 2a compares the clinical OS rates in the severe and moderate weight loss groups. The 5-year OS rates after oesophagectomy were 67% in the moderate weight loss group and 54% in the severe weight loss group, respectively. Hence, the OS rate in the severe weight loss group was significantly lower (*p* = 0.024) than that in the moderate weight loss group. The 5-year cancer-specific survival (CSS) rates were 71% in the moderate weight loss group and 61% in the severe weight loss group, and the CSS rates significantly in the severe weight loss group was lower than that in the moderate weight loss group (*p* = 0.039; Fig. 2b). The 5-year progression-free survival (PFS) rates were 60% in the moderate weight loss group and 49% in the severe weight loss group, the PFS rates were also lower in the severe weight loss group, but this difference did not reach significance (*p* = 0.068; Fig. 2c).

Previous study reported 10% weight loss was associated with poor prognosis after oesophagectomy [22, 23]. We compared clinical features and OS rates between patients with more than 10% weight loss (184 patients) and those with less than 10% weight loss (133 patients). No significant differences were found between 2 groups with respect to baseline characteristics and OS rates (Additional file 1: S-Table 1 and Additional file 2: S-Fig. 1).

### Univariate and multivariate analyses and risk factors for OS (Table 2)

**Table 2:** Univariate and multivariate Cox regression analyses for overall survival

Variables	Univariate analysis			Multivariate analysis		
	HR	95% CI	*Pp*	HR	95% CI	*p*
Age (≥ 75/ < 75)	1.02	0.48–2.19	0.96			
Sex (male/female)	1.20	0.61–2.39	0.59			
pDepth of tumor invasion (pT3-4/T1-2)	1.80	1.08–2.98	0.024^a^	1.89	1.12–3.17	0.016^a^
pLymph node metastasis (+/−)	1.37	0.86–2.19	0.17			
Residual tumor (+/−)	1.12	0.52–2.40	0.76			
Preoperative therapy (+/−)	2.03	1.22–3.39	0.0064^a^	2.11	1.25–3.54	0.0048^a^
Severe weight loss (+/−)	1.79	1.03–3.14	0.039^a^	1.82	1.02–3.24	0.039^a^
Anastomosis leakage (+/−)*	1.54	0.75–3.16	0.22			
Pulmonary complication (+/−)*	1.61	0.61–4.22	0.32^a^			
Recurrent nerve palsy (+/−)*	1.73	0.46–6.43	0.41			

Data were analyzed using logistic regression analysis

*HR* hazard ratio, *CI* confidence interval

^*^Postoperative morbidities were analyzed according to the Clavien–Dindo classification (3 and 4)

^a^ Statistically significant

The variables using univariate analyses are as follows: age, gender, pathological depth of tumor invasion, pathological lymph node metastasis, residual tumor, preoperative therapy, severe weight loss, anastomosis leakage, pulmonary complication and recurrent nerve palsy. The number of older patients (≥ 75) are 33 (10%), those of male patients are 277 (87%), those of patients with pathological deep tumor invasion (T3-4) are 113 (36%), those of patients with pathological lymph node metastasis positive are 162 (36%), those of patients with residual tumor 35 (11%), those of patients receiving preoperative therapy are 201 (63%), those of patients underwent anastomosis leakage 35 (11%), those of patients underwent pulmonary complications 18 (6%) and those of patients underwent recurrent nerve palsy are 13 (4%). Univariate analyses of predictors showed that the tumour invasion depth (pT3-4), preoperative therapy, and severe weight loss were associated with poor prognosis (*p* = 0.024, *p* = 0.0064 and *p* = 0.039, respectively). In the multivariate analysis, the deeper depth of tumor invasion group, preoperative therapy group and severe weight loss group had a worse overall survivals (hazard ratio = 1.89; 95% confidence interval = 1.12–3.17, hazard ratio = 2.11; 95% confidence interval = 1.25–3.54, hazard ratio = 1.82; 95% confidence interval = 1.02–3.524, respectively). No other predictors were significant in the univariate and multivariate analyses.

### Baseline characteristics of patients with FT placement

We assessed whether enteral nutrition via FT during oesophagectomy improved the OS of patients with oesophageal cancer. After classifying the patients into the FT group (n = 184) and no-FT group (n = 133), baseline clinical characteristics were adjusted by propensity score matching, which was employed for assembling two comparable groups. The following covariates were used for propensity score-matched analysis: age, sex, clinical tumour invasion depth, clinical lymph node metastasis, and preoperative therapy.

Table 3 summarises the demographic and clinical characteristics of the groups before and after propensity score matching. In the entire cohort, the FT and no-FT groups were comparable in terms of age, sex, clinical tumour location, preoperative body weight, anastomotic leakage, pulmonary complication, and recurrent nerve palsy. The percentage of deep tumour invasion (cT3-4) and the number of patients who underwent preoperative therapy were higher in the FT group than in the no-FT group (*p* = 0.044 and *p* = 0.0001, respectively). After propensity score matching (n = 119 per group), no significant differences were found between the FT and no-FT groups with respect to baseline characteristics.

**Table 3:** Baseline characteristics of the patients before (entire cohort) and after propensity score matching (matched cohort)

	Whole cohort		*p* value^a^	Matched cohort		*p* value^a^
	FT group (n = 184)	No-FT group (n = 133)		FT group (n = 119)	No-FT group (n = 119)	
Elderly patients (> 75)	23 (13%)	10 (8%)	0.14^b^	5 (4%)	6 (5%)	0.56^b^
Gender (%)			0.45^b^			0.70^b^
Male	163 (89%)	114 (86%)		104 (87%)	102 (86%)	
Female	23 (11%)	21 (14%)		14 (13%)	15 (14%)	
Tumor location			0.33^b^			0.26^b^
Ut/Mt/Lt	31/86/67	30/53/50		19/56/44	29/49/41	
Depth of tumor invasion			0.044^b^			0.24^b^
cT1-2	107 (58%)	92 (69%)		91 (76%)	83 (69%)	
cT3-4	77 (42%)	41 (36%)		28 (24%)	36 (31%)	
Lymph node metastasis (cN+)	101 (55%)	64 (48%)	0.23^b^	57 (48%)	56 (47%)	0.89^b^
Preoperative therapy (+)	133 (72%)	68 (51%)	0.0001^c^	49 (41%)	52 (44%)	0.69^b^
Preoperative body weight (kg)	56 (32–80)	58 (36–85)	0.21 ^c^	57 (32–79)	58 (36–85)	0.87^c^
Anastomotic leakage* (+)	22 (12%)	13 (9.8%)	0.53	17 (14%)	11 (9.2%)	0.23
Pulmonary complication* (+)	11 (6%)	7 (5.3%)	0.79	4 (3.4%)	4 (3.4%)	1.00
Recurrent nerve palsy* (+)	5 (2.7%)	8 (6%)	0.14	2 (1.7%)	6 (5%)	0.14

*FT* feeding jejunostomy tube

^*^Postoperative morbidity was analyzed according to the Clavien–Dindo classification (3 and 4)

^a^Comparison between the FT and no-FT groups

^b^χ^2^ test

^c^Student’s *t*-test

### Clinical outcomes in the FT and no-FT groups

**Fig. 3:**
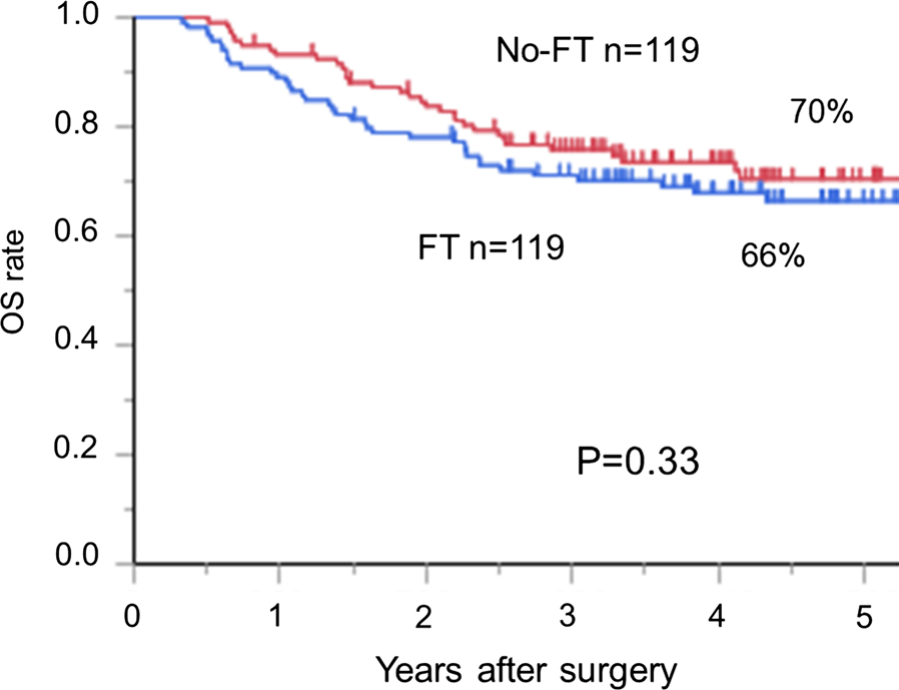
No differences in the overall survival (OS) rates were observed between the FT group (blue line) and the no-FT group (red line)

Table 4 and Fig. 3 compare the clinical outcomes in the propensity score-matched cohort. No significant differences were observed between the two groups in terms of postoperative weight loss and the number of patients with postoperative severe weight loss. The median duration of the FT placement was 78 (27–462) days, and 102 patients (86%) underwent home enteral feeding after hospital discharge. For the no-FT group, we did not give oral enteral supplement after oesophagectomy. For 9 patients in no-FT group, a nasojejunal tube was endoscopically placed after oesophagectomy in the entire cohort. The 5-year OS rates after oesophagectomy were 66% in the FT group and 70% in the no-FT group, respectively. Notably, enteral nutrition via FT could not improve the OS.

**Table 4:** Surgical outcomes in the propensity score-matched cohort

	FT group (n = 119)	No-FT group (n = 119)	*p* value
Weight loss (range)	12% (0–33)	10% (0–34)	0.25^a^
Patients with severe weight loss	26 (22%)	22 (18%)	0.52^b^
Duration of FT placement (days)*	78 (27–462)	-	-

*FT* feeding jejunostomy tube

^*^Data are expressed as median (range)

^a^Student’s *t*-test

^b^χ^2^ test

## Discussion

This study revealed that severe weight loss after MIO is significantly associated with poor OS and CSS in patients with oesophageal cancer.

Oesophagectomy for oesophageal cancer is still a highly morbid procedure, and postoperative malnutrition is commonly experienced, given that patients often fail to meet their caloric requirements [3]. Particularly, weight loss of 10%–15% of the body weight is observed in half of the patients after oesophagectomy[24]. Scarpa et al. and Wu et al. reported that MIO prevents the decrease in postoperative nutritional status [8, 9]. However, some patients substantially lost their body weight after MIO [25, 26]. The present study demonstrated the association between postoperative severe weight loss and long-term prognosis after MIO.

Hynes et al. and D’Journo et al. stated that postoperative weight loss had a significant effect on the OS of 390 and 205 patients with oesophageal cancer, respectively [22, 27]. These two studies evaluated weight loss at 6 [27] and 12 months [22] after oesophagectomy. Okada et al. reported that the degree of postoperative weight loss is most severe in the first 3 months after oesophagectomy [28]. The effect of weight loss after MIO on long-term prognosis has remained under-investigated. In this study, we evaluated postoperative weight loss at 3 months after MIO.

Previous studies reported that 1-year postoperative malnutrition was defined as a 10% weight loss according to the WHO definition[22, 29]. D’Journo et al. [22] reported postoperative weight loss of more than 10% might affect survival after esophagectomy for oesophageal cancer. In this study, 60% of patients experienced more than 10% weight loss. No significant differences were found between these two groups. We might not have conducted appropriate nutritional support during the oesophagectomies. For FT group, we could not demonstrate improvement of postoperative weight loss. But some studies reported FT improved nutritional status [30, 31]. In the FT group, the median duration of FT placement was 78 days, and 102 patients (86%) underwent home enteral feeding after hospital discharge. Weijis et al. reported that weight loss following oesophagectomy occurs once tube feeding is stopped [32]. Nutritional support via FT may be needed for a longer time after oesophagectomy. The consideration of proper enteral nutrition protocol may be needed. Enteral nutrition with enriched eicosapentaenoic acid (EPA) preserves lean body mass after esophagectomy [33]. Another study reported that an enteral diet enriched with ω-3 fatty acids improves oxygenation after thoracic oesophagectomy [32]. Appropriate nutritional support may prevent malnutrition of the patients after oesophagectomy. For the no-FT group, we did not give nutritional support. This might suggest that they should take an oral nutritional supplement. Our results suggest that Improvement of nutritional support for the FT group and no-FT group was needed to reduce postoperative weight loss and improve outcomes.

To date, to our best knowledge, no studies have assessed whether these nutritional interventions can improve long-term prognosis after oesophagectomy. In this study, we could not demonstrate that enteral nutrition via FT improved the incidence of severe weight loss and the OS in patients after MIO. Proper enteral nutrition protocol, including the contents, amounts and duration of an enteral nutrition, might need to be considered. Appropriate nutritional support might prevent malnutrition in patients who underwent oesophagectomy. Okada et al. stated that the adequacy of postoperative oral intake after esophagectomy affects the postoperative nutritional status and the OS [4]. Patients with poor oral intake fulfilled their nutritional requirements by enteral nutrition via FT but still had poor prognoses [4]. It might be necessary to improve the adequacy of oral intake after esophagectomy, and oral intake might be given priority over enteral nutrition via FT and parenteral nutrition.

In this study, the deeper depth of tumor invasion was associated with poor prognosis, but pathological lymph node metastasis and residual tumor status were not. Receiving preoperative therapy was independent risk factor of poor prognosis. We performed preoperative therapy for patients with cT2-4 or clinically lymph node positive. These clinical statuses might be strongly associated with poor prognosis than pathological lymph node metastasis.

Meanwhile, this study has several limitations. No difference in the OS rates were observed between the FT group and no-FT group after MIO. This is a small sample size and retrospective study. We attempted to adjust differences in the characteristics of patients using propensity score matching. However, it is not a substitute for randomisation. Further studies, including prospective randomized studies for the patients with and without FT are needed. In this study, the association between postoperative severe weight loss and long-term prognosis after MIO was demonstrated. Previous studies reported that MIO prevents the decrease in postoperative nutritional status [8, 9] and decreases postoperative complication [34, 35]. MIO might be different a procedure from open oesophagectomy. Large number studies including two cohort, open esophagectomy and MIO, should be conducted. In this study, there were no criteria about FT placement. FT placement was decided by the attending surgeon. Patients with obstructing oesophageal carcinomas were administered oral nutritional supplements via a nasojejunal tube during preoperative therapy. FT was routinely placed for them. Therefore, FT might be likely placed in patients with deep tumour invasion and those who had undergone preoperative therapy. To determine the effects of FT placement on weight loss and survival, further studies are needed to identify the patients in whom FT is essential.

Park et al. and Mayanagi et al. reported decreased skeletal muscle index (SMI) in the psoas muscle area after oesophagectomy in 58 and 66 patients, respectively, thereby leading to a negative prognostic effect on OS. However, they reported that weight loss after oesophagectomy had no significant effect on OS [5, 6], even though BMI and SMI are strongly and directly related [36, 37]. SMI reduction might be a clearer indicator than BMI reduction for the prediction of oesophageal cancer prognosis. However, the measurement of body weight is more objective and convenient than that of SMI. Above all, patients can measure their body weight by themselves. In this study, we evaluated postoperative weight loss at 3 months after MIO, and the severe weight loss cut off point was 17%. If we investigate other cutoff point and nutritional index, including SMI and serum albumin at different timings (6 months and 12 months postoperatively), we may get different results. Further studies with larger numbers are needed.

## Conclusion

Postoperative severe weight loss after MIO in a prone position is significantly associated with poor survival in patients with oesophageal cancer, and enteral nutrition via FT could not improve severe weight loss, thereby leading to poor survival.

## Supplementary information

**Additional file 1: S-Table 1.** Comparison of clinical features between the more than 10% weight loss and less than 10% weight loss groups.

**Additional file 2: S-Fig. 1.** No differences in the overall survival (OS) rates were observed between the more than10% weight loss group (blue line) and the more than 10% weight loss group (red line).

## Data Availability

The datasets used and/or analysed during the current study available from the corresponding author on reasonable request.

## Abbreviations

FT: Feeding jejunostomy tube
MIO: Minimally invasive oesophagectomy
OS: Overall survival
BMI: Body mass index
CT: Computed tomography
HR: Hazard ratio
CI: Confidence interval

## References

[1] Ferlay J, Soerjomataram I, Dikshit R (2015). Cancer incidence and mortality worldwide: sources, methods and major pattern in GLOBOCAN 2012. Int J Cancer.

[2] Morita M, Yoshida R, Ikeda K (2008). Advances in esophageal cancer surgery in Japan: an analysis of 1000 consecutive patients treated at a single institute. Surgery.

[3] Baker M, Halliday V, Williams RN (2016). A systematic review of the nutritional consequences of esophagectomy. Clin Nutr.

[4] Okada G, Momoki C, Habu D (2019). Effect of postoperative oral intake on prognosis for esophageal cancer. Nutrients.

[5] Park SY, Yoon JK, Lee SJ (2017). Postoperative change of the psoas muscle area as a predictor of survival in surgically treated esophageal cancer patients. J Thorac Dis.

[6] Mayanagi S, Tsubosa Y, Omae K (2017). Negative impact of skeletal muscle wasting after neoadjuvant chemotherapy followed by surgery on survival for patients with thoracic esophageal cancer. Ann Surg Oncol.

[7] Yamashita K, Watanabe M, Mine S (2018). Minimally invasive esophagectomy attenuates the postoperative inflammatory and improves survival compared with open esophagectomy in patients with esophageal cancer: a propensity score matched analysis. Surg Endosc.

[8] Scarpa M, Cavallin F, Saaddeh LM (2016). Hybrid minimally invasive esophagectomy for cancer: impact on postoperative inflammatory and nutritional status. Dis Esophagus.

[9] Wu Z, Wu M, Wang Q (2018). Home enteral nutrition after minimally invasive esophagectomy can improve quality of life and reduce the risk of malnutrition. Asia Pac J Clin Nutr.

[10] Barlow R, Price P, Reid TD (2011). Prospective multicentre randomised controlled trial of early enteral nutrition for patients undergoing major upper gastrointestinal surgical resection. Clin Nutr.

[11] Xiao-Bo Y, Qiang L, Xiong Q (2014). Efficacy of early postoperative enteral nutrition in supporting patients after esophagectomy. Minerva Chir.

[12] Findlay JM, Gillies RS, Millo J (2014). Enhanced recovery for esophagectomy: a systematic review and evidence-based guidelines. Ann Surg.

[13] Sobin LH, Gospodarowicz MK, Wittekind C (2011). TNM classification of malignant tumors.

[14] Ando N, Kato H, Igaki H (2012). A randomized trial comparing postoperative adjuvant chemotherapy with cisplatin and 5-fluorouracil versus preoperative chemotherapy for localized advanced squamous cell carcinoma of the thoracic esophagus (JCOG9907). Ann Surg Oncol.

[15] Oshikiri T, Yasuda T, Harada H (2015). A new method (the “Bascule method”) for lymphadenectomy along the left recurrent laryngeal nerve during prone esophagectomy for esophageal cancer. Surg Endosc.

[16] Oshikiri T, Nakamura T, Hasegawa H (2018). Standardizing procedures improve and homogenizes short-term outcomes after minimally invasive esophagectomy. Langenbecks Arch Surg.

[17] Oshikiri T, Yasuda T, Kawasaki K (2016). Hand-assisted laparoscopic surgery (HALS) is associated with less-restrictive ventilatory impairment and less risk for pulmonary complication than open laparotomy in thoracoscopic esophagectomy. Surgery.

[18] Galon J, Costes A, Sanchez-Cabo F (2006). Type, density, and location of immune cells within human colorectal tumors predict clinical outcome. Science.

[19] He X, Li JP, Liu XH (2018). Prognostic value of C-reactive protein/albumin ratio in predicting overall survival of Chinese cervical cancer patients overall survival: comparison among various inflammation based factors. J Cancer.

[20] Rosenbaum PR, Rubin DB (1983). The central role of the propensity score in observational studies for causal effects. Biometrika.

[21] Clavien PA, Barkun J, de Oliveria ML (2009). The Clavien–Dindo classification of surgical complication: five-year experience. Ann Surg.

[22] D’Journo XB, Ouattara M, Loudou A (2012). Prognostic impact of weight loss in 1-year survivors after transthoracic esophagectomy for cancer. Dis Esophagus.

[23] Kitagawa H, Namikawa T, Munekage M (2016). Analysis of factors associated with weight loss after esophagectomy for esophageal cancer. Anticancer Res.

[24] Martin L, Lagergren J, Lindblad M (2007). Malnutrition after oesophageal cancer surgery in Sweden. Br J Surg.

[25] Wang P, Li Y, Sun H (2019). Analysis of the associated factors for weight loss after minimally invasive Mckeown esophagectomy. Thorac Cancer.

[26] Park SY, Kim DJ, Suh JW (2018). Risk factors for weight loss 1 year after esophagectomy and gastric pull-up for esophageal cancer. J Gastrointest Surg.

[27] Hynes O, Anandavadivelan P, Gossage J (2017). The impact of pre- and post-operative weight loss and body mass index on prognosis in patients with esophageal cancer. Eur J Surg Oncol.

[28] Okada G, Matsumoto Y, Nakamura Y (2017). Nutritional changes and factors contributing to postoperative weight recovery after esophagectomy. Esophagus.

[29] Obesity: preventing and managing the global epidemic. Report of a WHO consultation. World Health Organ Tech Rep Ser. 2000;894:1–253. 11234459

[30] Takesue T, Takeuchi H, Ogura M (2015). A Prospective randomized trial of enteral nutrition after thoracoscopic esophagectomy for esophageal cancer. Ann Surg Oncol.

[31] Weijs TJ, van Eden HWJ, Ruurda JP (2017). Routine jejunostomy tube feeding following esophagectomy. J Thorac Dis.

[32] Ryan AM, Reynolds JV, Healy L (2009). Enteral nutrition enriched with eicosapentaenoic acid (EPA) preserves lean body mass following esophageal cancer surgery:results of a double-blinded randomized controlled trial. Ann Surg.

[33] Matsuda Y, Habu D, Lee S (2017). Enteral diet enriched with ω-3 fatty acid improves oxygenation after thoracic esophagectomy. World J Surg.

[34] Navidi M, Phillips AW (2019). Hybrid minimally invasive esophagectomy for esophageal cancer. N Engl J Med.

[35] Shen Y, Zhong M, Wu W (2013). The impact of tidal volume on pulmonary complications following minimally invasive esophagectomy: a randomized and controlled study. J Thorac Cardiovasc Surg.

[36] Hervochon R, Bobbio A, Guinet C (2017). Body mass index and total psoas area affect outcomes in patients undergoing pneumonectomy for cancer. Ann Thorac Surg.

[37] Hammad A, Kaido T, Hamaguchi Y (2017). Impact of sarcopenic overweight on the outcomes after living donor liver transplantation. Hepatob Surg Nutr.

