# Supplementary material for: Severe weight loss after minimally invasive oesophagectomy is associated with poor survival in patients with oesophageal cancer at 5 years
Source: BMC Gastroenterol. 2020 Dec 3;20:407. doi: 10.1186/s12876-020-01543-1 (PMC7713340; doi:10.1186/s12876-020-01543-1)
Supplement: Supplementary file 1 — Additional file 1: S-Table 1. Comparison of clinical features between the more than 10% weight loss and less than 10% weight loss groups. [file 12876_2020_1543_MOESM1_ESM.docx]

Additional file 2: S-Table 1. Comparison of clinical features between the more than 10% weight loss and less than 10% weight loss groups

|  | More than 10% weight  loss (n = 184) | Less than 10% weight  loss (n = 133) | P  value |
| --- | --- | --- | --- |
| Age (years) * | 66 (43-82) | 65 (27-80) | 0.11 ^a^ |
| Gender (%) |  |  | 0.94 ^b^ |
| Male | 161 (88%) | 116 (87%) |  |
| Female | 23 (12%) | 17 (13%) |  |
| Location of tumor |  |  | 0.68 ^b^ |
| Ut/Mt/Lt | 36/77/71 | 25/62/46 |  |
| clinical Depth of tumor invasion |  |  | 0.11 ^b^ |
| cT1/T2/T3/T4 | 20/23/22/0 | 101/55/93/3 |  |
| clinical Lymph node metastasis |  |  | 0.42 ^b^ |
| cN0/N1/N2/N3 | 25/32/7/0 | 125/102/25/1 |  |
| Preoperative therapy (+) | 122 (66%) | 79 (49%) | 0.21 ^b^ |
| pathological Depth of tumor invasion |  |  | 0.39 ^b^ |
| pT1/T2/T3/T4 | 100/16/63/5 | 70/17/45/1 |  |
| pathological Lymph node metastasis |  |  | 0.11 ^b^ |
| pN0/N1/N2/N3 | 91/55/21/17 | 64/46/19/4 |  |
| Residual tumor |  |  | 0.65 ^b^ |
| R0/R1/R2 | 165/15/4 | 116/15/2 |  |
| Preoperative body weight (kg) * | 58 (35-85) | 56 (32-83) | 0.17 ^a^ |
| Anastomotic leakage** (+) | 23 (%) | 12 (%) | 0.33 ^b^ |
| Pulmonary complication** (+) | 11 (%) | 7 (%) | 0.79 ^b^ |
| Recurrent nerve palsy** (+) | 7 (%) | 6 (%) | 0.76 ^b^ |

*Data are expressed as the median (range).

FJT: feeding jejunostomy tube

**Postoperative morbidity was analyzed according to the Clavien–Dindo classification (3 and 4).

^a^ χ2 test

^b^ Student’s *t*-test
