# Supplementary figures and images for: Severe weight loss after minimally invasive oesophagectomy is associated with poor survival in patients with oesophageal cancer at 5 years
Source: BMC Gastroenterol. 2020 Dec 3;20:407. doi: 10.1186/s12876-020-01543-1 (PMC7713340; doi:10.1186/s12876-020-01543-1)

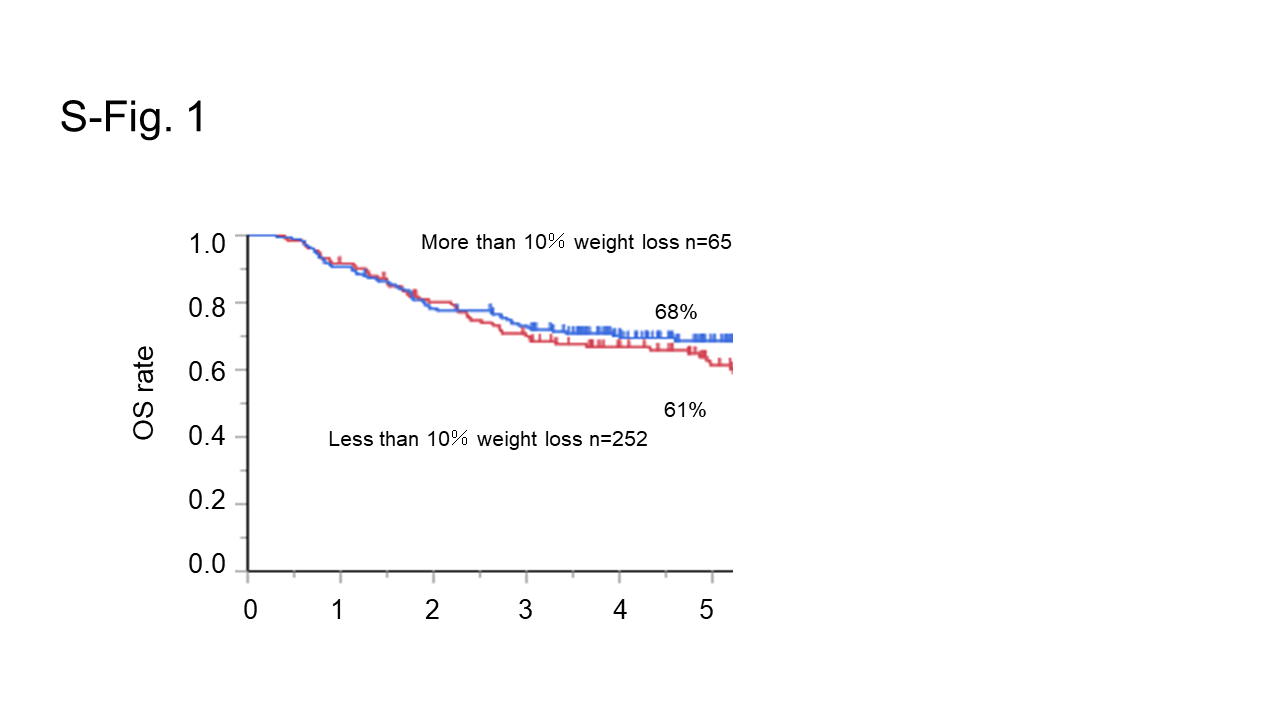

Supplement: Supplementary file 2 — Additional file 2: S-Fig. 1. No differences in the overall survival (OS) rates were observed between the more than10% weight loss group (blue line) and the more than 10% weight loss group (red line). [file 12876_2020_1543_MOESM2_ESM.tif]
